# Predicting Treatment Interruption Among People Living With HIV in Nigeria: Machine Learning Approach

**DOI:** 10.2196/44432

**Published:** 2023-05-12

**Authors:** Matthew-David Ogbechie, Christa Fischer Walker, Mu-Tien Lee, Amina Abba Gana, Abimbola Oduola, Augustine Idemudia, Matthew Edor, Emily Lark Harris, Jessica Stephens, Xiaoming Gao, Pai-Lien Chen, Navindra Etwaroo Persaud

**Affiliations:** 1 FHI 360 Abuja Nigeria; 2 FHI 360 Washington, DC United States; 3 FHI 360 Durham, NC United States; 4 United States Agency for International Development Dar es Salaam United Republic of Tanzania; 5 United States Agency for International Development Washington, DC United States

**Keywords:** HIV, machine learning, treatment interruption, Nigeria, chronic disease, antiretroviral therapy, chronic disease, HIV program, intervention, data collection

## Abstract

**Background:**

Antiretroviral therapy (ART) has transformed HIV from a fatal illness to a chronic disease. Given the high rate of treatment interruptions, HIV programs use a range of approaches to support individuals in adhering to ART and in re-engaging those who interrupt treatment. These interventions can often be time-consuming and costly, and thus providing for all may not be sustainable.

**Objective:**

This study aims to describe our experiences developing a machine learning (ML) model to predict interruption in treatment (IIT) at 30 days among people living with HIV newly enrolled on ART in Nigeria and our integration of the model into the routine information system. In addition, we collected health workers’ perceptions and use of the model’s outputs for case management.

**Methods:**

Routine program data collected from January 2005 through February 2021 was used to train and test an ML model (boosting tree and Extreme Gradient Boosting) to predict future IIT. Data were randomly sampled using an 80/20 split into training and test data sets, respectively. Model performance was estimated using sensitivity, specificity, and positive and negative predictive values. Variables considered to be highly associated with treatment interruption were preselected by a group of HIV prevention researchers, program experts, and biostatisticians for inclusion in the model. Individuals were defined as having IIT if they were provided a 30-day supply of antiretrovirals but did not return for a refill within 28 days of their scheduled follow-up visit date. Outputs from the ML model were shared weekly with health care workers at selected facilities.

**Results:**

After data cleaning, complete data for 136,747 clients were used for the analysis. The percentage of IIT cases decreased from 58.6% (36,663/61,864) before 2017 to 14.2% (3690/28,046) from October 2019 through February 2021. Overall IIT was higher among clients who were sicker at enrollment. Other factors that were significantly associated with IIT included pregnancy and breastfeeding status and facility characteristics (location, service level, and service type). Several models were initially developed; the selected model had a sensitivity of 81%, specificity of 88%, positive predictive value of 83%, and negative predictive value of 87%, and was successfully integrated into the national electronic medical records database. During field-testing, the majority of users reported that an IIT prediction tool could lead to proactive steps for preventing IIT and improving patient outcomes.

**Conclusions:**

High-performing ML models to identify patients with HIV at risk of IIT can be developed using routinely collected service delivery data and integrated into routine health management information systems. Machine learning can improve the targeting of interventions through differentiated models of care before patients interrupt treatment, resulting in increased cost-effectiveness and improved patient outcomes.

## Introduction

Antiretroviral therapy (ART) for HIV treatment has transformed HIV from a fatal illness to a lifelong, yet manageable, chronic disease [1]. Long-term adherence to ART and subsequent viral load suppression decrease morbidity and mortality, and reduce the risk of viral transmission [2]. As increasing numbers of countries meet the United Nations Joint Programme on HIV/AIDS (UNAIDS) 95-95-95 benchmarks, tailored interventions and data systems are needed to proactively identify the individuals at highest risk and reduce interruption in treatment (IIT) to achieve and sustain epidemic control [3]. Such data and systems must reflect the reality that retention is not a linear pathway; instead, patients cycle in and out of care. Data from the US President’s Emergency Plan for AIDS Relief (PEPFAR) for the period from January 1 to March 31, 2022, show that approximately 4.8% of all patients on ART cycle in and out of treatment (US President’s Emergency Plan for AIDS Relief, unpublished data, March 2023). Historically, data from sub-Saharan Africa have suggested that the proportion of individuals remaining on HIV therapy after 3 years has been about 65% [4].

HIV programs use a range of programmatic approaches to support individuals in sustaining adherence to ART and re-engaging those who interrupt treatment [5]. These interventions for preventing IIT or re-engaging those who have already interrupted their treatment can be time-consuming and costly if not targeted. This can lead to inefficiencies from public health, resource management, and sustainability perspectives [6,7]. Innovative approaches to identifying individuals at high risk of IIT and tailored activities to prevent IIT are needed to ensure optimal client health and sustained epidemic control [8,9]. Applying machine learning (ML) for predicting individuals at high risk of IIT paves the way for differentiated service delivery solutions that are individualized, evidence-based, and responsive to improve retention in care and treatment in the path toward epidemic control.

Large data sets containing individual-level data for people living with HIV are now widely available and may create new opportunities to identify patterns and relationships between individual factors and observed client outcomes. Mathematical models can take the process a step further and use retrospective data to predict future behavior [10]. This application of ML is part of a broader trend leveraging artificial intelligence across a range of development sectors, including agriculture, health, and natural disaster response systems [11,12]. HIV use cases have been developed to understand how predictive analytics can improve client services and reduce service delivery pain points across the HIV continuum of care. These use cases enhance our understanding of the theory of change for how predictive analytics can improve HIV clinical outcomes, program efficiency, and cost-effectiveness. One of the use cases developed in South Africa, termed the “Fall-Out Forecaster,” models how recognizing client risk factors can lead to optimized treatment support interventions and minimize IIT. This model could reduce IIT by 6%-10% and reduce care and support costs by 4%-5% in the first 12 months [13].

The real-world application of theoretical HIV use cases of ML in low- and middle-income settings is growing. In Nigeria and Kenya, ML was applied to retrospective patient-level data sets. The models identified independent predictors of IIT among patients receiving ART in Kenya and helped create behavioral risk profiles [14]. In South Africa, retrospective data for clinical, laboratory, and visit patterns were used to develop an ML algorithm that identifies individuals at risk of unsuppressed viral load at their next visit [15]. In Haiti, health care workers used an ML algorithm to generate client risk scores that classified clients into five categories of risk for treatment failure [16]. Health care workers were subsequently trained to provide culturally sensitive, tailored psychosocial counseling to promote retention among clients assessed as high-risk. In South Africa, an ML model helped to define a unique set of retention services tailored for each client [17,18]. In Mozambique, efforts starting in 2018 used ML models to generate risk scores for client likelihood of interrupting treatment (integrated into service delivery via a mobile app or an OpenMRS “plug-in”); the integration demonstrated the ability to rank clients by overall risk, but the ability to plan treatment retention services according to risk level is still under study [19].

In this paper, we describe the development of an ML model to predict IIT at 30 days among people living with HIV newly enrolled in ART in Nigeria and our experiences integrating the model into the routine HIV treatment program. We report the process of model development, early experiences integrating the model into a routine health management information system, and ML users’ perceptions and use of the model outputs for case management.

## Methods

### Program Description

The Strengthening Integrated Delivery of HIV/AIDS Services (SIDHAS) project in Nigeria supports the government of Nigeria in implementing comprehensive HIV services in Akwa Ibom and Cross River states. The goal is to sustain the integration of HIV and AIDS services with tuberculosis (TB) services by building the capacity of the government of Nigeria staff to deliver high-quality, comprehensive, preventive care and treatment and other related services. The project, which began in 2011, currently supports treatment at 154 health facilities including public, private for-profit, and faith-based organizations; 103 community pharmacies; and 2684 other community ART refill structures. To support case management, individual-level client data are recorded in the electronic medical record system, Lafiya Management Information System (LAMIS).

### Data Collection and Cleaning

For this study, we used routine program data from the SIDHAS project to quantify the association of individual characteristics with IIT among people living with HIV receiving ART and developed an ML model to predict future IIT. Data from the patient, clinic, and pharmacy data sets from Akwa Ibom and Cross River states in Nigeria collected from January 2005 through February 2021 were extracted from LAMIS and used for model development. These service delivery data are collected using standardized paper-based forms at each patient encounter and then entered into LAMIS by facility staff. All personal identifiers were removed, and patient data were linked to create one consolidated data set using the unique treatment identification number. We included all patients who were newly enrolled on ART and provided a 30-day supply of antiretrovirals (ARVs) at one of the SIDHAS-supported treatment facilities. The three separate databases were reviewed, and data for selected variables were extracted for all eligible individuals. For the purposes of the study, individuals were defined as having IIT if they were provided a 30-day supply of ARVs but did not return for a refill within 28 days of their scheduled follow-up visit date.

The consolidated data set was subjected to a series of internal consistency checks during which records with invalid data were removed. Reasons for record removal included that the ART start date was listed as earlier than the date of the confirmed HIV test, participants were enrolled too recently to have an observed end point, and the date of the next appointment after enrollment was missing. Participants who were transferred in from other facilities were also excluded given that the interest was in IIT after ART initiation.

Missing data were then addressed for the remaining records in the cleaned data set. Two approaches were used to handle missing data based on the nature of the data collection and operation in the program field. First, missing data within the patient data set were imputed using the k-nearest neighbor algorithm [20] in which the missing value was classified by a plurality vote of its neighbors and the class most common among its k-nearest neighbors was assigned. Second, for variables such as TB status that could not be imputed, missing data within the clinic data set were classified as “missing” in the final data set. In addition, variables such as pregnancy/breastfeeding status for male clients or female clients younger than 10 years or older than 60 years that had incorrect values were categorized as “not applicable.”

### Variable Selection

The predictor variables that were used for model building were extracted from the routine health information system. They were preselected as they were considered to be strongly associated with treatment interruption by a group of SIDHAS project staff and HIV prevention and treatment experts in consultation with biostatisticians. The variables selected for the model included age, gender, marital status, occupation, education, local government area, baseline clinic stage, TB status, pregnancy and breastfeeding status, and facility characteristics (service level, facility type, ownership, population setting, state, ward, and care entry point). The feature (predictor) importance was applied to understand the data and to improve model building and interpretability.

### Model Development, Validation, and Testing

The final cleaned data set was randomly divided into a training data set containing 80% of the clients and a test data set with the remaining 20% of the clients. The first data set was used to train predictive models using the 10-fold cross-validation approach, while the second was used to validate model performance. Boosting classification algorithms (eg, boosting tree and Extreme Gradient Boosting) were applied to build predictive models. Positive predictive value, negative predictive value, and Cohen kappa were used to assess the performance of predictive models. The models were further validated on a second data set containing 1107 clients who initiated ART from March through October 2021.

### Field Implementation and User Experience

A total of 10 pilot sites were selected for field-testing of the ML model. These sites included primary, secondary, and tertiary service delivery points with adequate patient volume to ensure adequate new client enrollment. The ML algorithm was programmed into LAMIS such that after data from each new patient were entered into the database, the person’s IIT chance was automatically generated. At the end of each week, a list that showed the risk of IIT among those provided with a 30-day supply of ARVs was generated and shared with facility staff. Project staff, health care workers, and treatment supporters at the 10 selected facilities were trained on the basics of ML and on the interpretation and application of IIT scores in patient management. Persons with an IIT score of 50% or more were considered to be at high risk for IIT and their case managers provided additional monitoring and assigned an expert to provide psychosocial support through virtual or physical mechanisms to ensure that the client was mentally prepared for the challenges of lifelong ART. All other persons received the standard case management support that is provided to all clients.

Feedback from the health care workers at the pilot sites was collected in two ways. First, we routinely gathered verbal feedback as part of “daily situation room meetings.” These standing meetings were designed to review routine data and gave health care workers a platform to ask questions about the scores, clarify how the tool was working, and contribute practical suggestions for improvement. Second, we collected user feedback formally using a Google Forms questionnaire. The questionnaire in Google Forms was distributed electronically to health care workers at the selected pilot facilities, and they provided written feedback. The form collected information on the sociodemographic characteristics of the respondents; usefulness, acceptance, and relevance of the ML outputs for improving patient care; experiences interpreting and using the ML scores; and any suggestions for improving the presentation of the scores. The data from the two sources were combined and summarized according to key themes.

### Ethical Considerations

The data for this study were collected from an existing project database that is used for routine patient management and program monitoring. The study was reviewed by the Protection of Human Subjects Committee at FHI 360 and was categorized as research not involving human subjects. The authors had no access to patients or personally identifiable information for the individuals whose data were included in the study.

## Results

### Model Development

**Figure 1:**
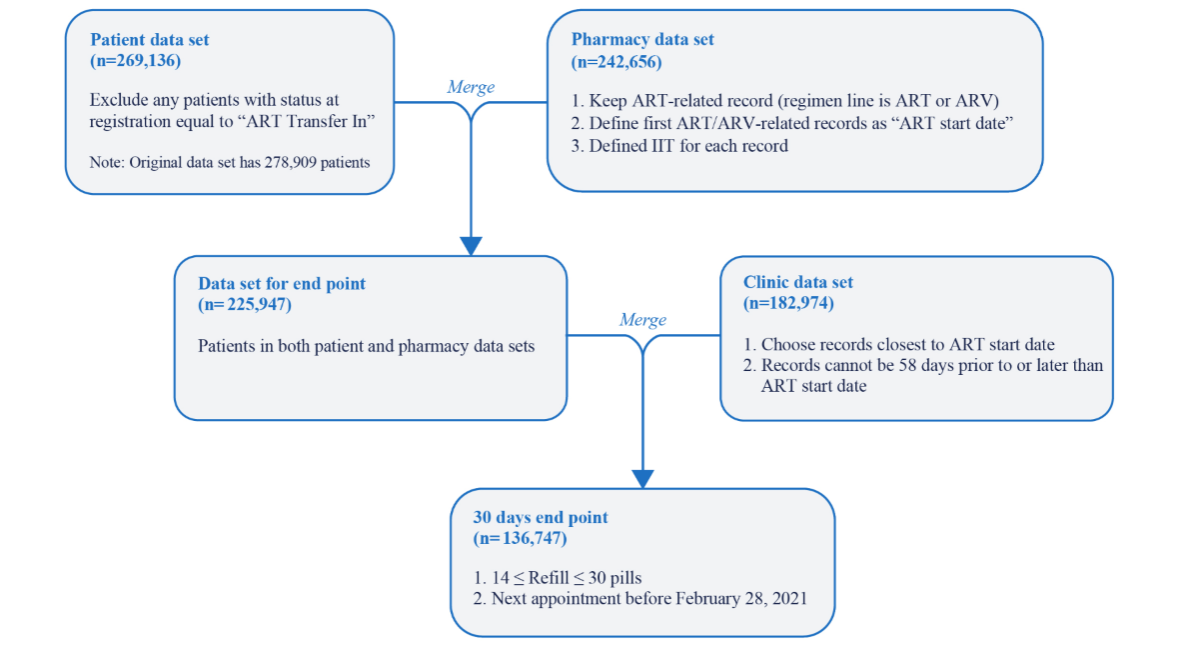
Study cohort flow diagram. ART: antiretroviral therapy; ARV: antiretroviral; IIT: interruption in treatment.

After data cleaning, complete data from a total of 136,747 clients were used for the analysis (Figure 1).

The percentage of IIT cases was 41.5% (56,581/136,747) overall but changed over time (Table 1). It decreased significantly during successive years, ranging from 58.6% (36,663/61,864) before 2017 to 14.2% (3690/28,046) during October 2019 through February 2021. Clients sicker at enrollment had higher IIT rates; IIT was 31.7% (20,465/64,508) among individuals with stage I disease at enrollment compared to 43.5% (12,867/29,557) among those with stage II disease and 59% (2125/3600) among those with stage IV disease. A greater proportion of clients whose baseline clinical stage or baseline clinic data (TB, pregnancy, and breastfeeding status) were missing were classified as IIT compared to individuals with data available for these variables. Other variables that were significantly associated with IIT rates were facility characteristics: location, service level, and service type. IIT rates did not vary significantly by age, gender, education level, marital status, or occupation.

To incorporate the features of the variables, eight models were trained using training data sets with and without year of ART initiation, clinic data (TB, pregnancy, and breastfeeding status), or facility characteristics. The results indicated that models without clinic data would lose more than 10% of predictive accuracy compared to those models with clinic data included, whereas the facility information and year of ART initiation variables only had a slight impact on model performance (Table 2). The results of the model testing on the data from March through October 2021 were similar to the results observed from the test data. These findings indicated that the predictive models were robust and useful for future IIT prediction in the same setting of ART programs.

**Table 1:** Characteristics of the individuals included in the data set used for the model development.

Variable and category		Individuals (N=136,747), n (%)
**Interruption in treatment**		
	Yes	56,581 (41.38)
	No	80,166 (58.62)
**Year of antiretroviral initiation**		
	Before 2017	61,939 (45.3)
	January 2017-September 2019	46,776 (34.2)
	October 2019-February 2021	28,032 (20.5)
**Gender**		
	Female	91,982 (67.26)
	Male	44,765 (32.74)
**Age (years)**		
	<14	5657 (4.14)
	14-20	8685 (6.35)
	21-35	72,049 (52.69)
	>35	50,356 (36.82)
**Marital status**		
	Married	1171 (0.86)
	Single	64,899 (47.46)
	Previously married	52,934 (38.71)
**Education**		
	Primary and Quranic	16,473 (12.05)
	≥1 year of secondary	44,219 (32.34)
	None	50,912 (37.23)
**Occupation**		
	Employed	36,863 (26.96)
	Unemployed/retired/students	79,059 (57.81)
**State**		
	Akwa Ibom	100,937 (73.82)
	Cross River	35,791 (26.18)
**Baseline clinic stage**		
	Stage I	64,508 (47.17)
	Stage II-IV	68,740 (50.27)
**Facility type**		
	Health center/clinic/posts	77,597 (56.75)
	General, tertiary, or cottage hospital	59,131 (43.25)
**TB^a^ status^b^**		
	No signs or symptoms of TB	58,953 (43.11)
	Currently on isoniazid prophylaxis	4745 (3.47)
	Confirmed/suspected TB	5167 (3.8)
**Pregnant^c^**		
	No	46,883 (51.0)
	Yes	925 (1.0)
**Breastfeeding^c^**		
	No	47,617 (51.8)
	Yes	191 (0.21)

^a^TB: tuberculosis.

^b^Totals do not add up to 136,747 for all variables under TB status due to missing values for some variables.

^c^n=91,982 (number of females in the data set).

**Table 2:** Model performance evaluation with test data from January 2005 through February 2021 and validation data for model 4.

	Model 1^a^	Model 2^b^	Model 3^c^	Model 4^d^ (selected)^e^	Model 4^f^	Model 5^g^	Model 6^h^	Model 7^i^	Model 8^j^
Accuracy (95% CI)	0.85 (0.85-0.86)	0.83 (0.83-0.84)	0.87 (0.87-0.87)	0.85 (0.85-0.86)	0.91 (0.88-0.93)	0.75 (0.74-0.75)	0.70 (0.69-0.70)	0.75 (0.74-0.75)	0.72 (0.72-0.73)
Sensitivity (95% CI)	0.82 (0.81-0.82)	0.75 (0.75-0.76)	0.84 (0.83-0.84)	0.81 (0.81-0.82)	0.79 (0.73-0.86)	0.63 (0.62-0.64)	0.58 (0.57-0.59)	0.63 (0.62-0.64)	0.62 (0.61-0.63)
Specificity (95% CI)	0.88 (0.87-0.88)	0.89 (0.88-0.89)	0.89 (0.89-0.90)	0.88 (0.88-0.89)	0.94 (0.92-0.96)	0.83 (0.82-0.84)	0.78 (0.77-0.78)	0.83 (0.82-0.84)	0.80 (0.79-0.81)
PPV^k^ (95% CI)	0.83 (0.82-0.83)	0.82 (0.82-0.83)	0.85 (0.84-0.85)	0.83 (0.82-0.83)	0.77 (0.70-0.83)	0.72 (0.72-0.73)	0.65 (0.64-0.66)	0.72 (0.71-0.73)	0.69 (0.68-0.70)
NPV^l^ (95% CI)	0.87 (0.87-0.88)	0.84 (0.83-0.84)	0.89 (0.88-0.89)	0.87 (0.87-0.88)	0.94 (0.93-0.96)	0.76 (0.76-0.77)	0.72 (0.72-0.73)	0.76 (0.76-0.77)	0.75 (0.74-0.75)
Kappa	0.69	0.65	0.73	0.70	0.72	0.47	0.36	0.47	0.42

^a^Model 1 included clinic variables (tuberculosis [TB], pregnancy, and breastfeeding status) and year of antiretroviral therapy (ART) initiation.

^b^Model 2 included clinic variables (TB, pregnancy, and breastfeeding status).

^c^Model 3 included clinic variables (TB, pregnancy, and breastfeeding status), facility information, and year of ART initiation.

^d^Model 4 included clinic variables (TB, pregnancy, and breastfeeding status) and facility information.

^e^Model selected for application.

^f^Validation data March-November 2021.

^g^Model 5 included year of ART initiation and did not include clinical variables (TB, pregnancy, and breastfeeding status).

^h^Model 6 did not include clinic variables (TB, pregnancy, and breastfeeding status), facility information, and year of ART initiation.

^i^Model 7 included facility information and year of ART initiation and did not include clinic variables (TB, pregnancy, and breastfeeding status).

^j^Model 8 included facility information and did not include clinic variables (TB, pregnancy, and breastfeeding status) and year of ART initiation.

^k^PPV: positive predictive value.

^l^NPV: negative predictive value.

### Field Implementation and User Experience

The 30-day predictive model was integrated into LAMIS and applied to 25 consecutive people living with HIV newly enrolled on ART at selected hospitals and who were provided with a 30-day supply of an ART regimen over a 15-week period (April to July 2022). None were seen to be a high risk for IIT based on the predetermined 50% threshold. The predicted IIT risks ranged from 1.8% to 25.7%. All clients received routine psychosocial support, monitoring of possible adverse drug reactions, and overall support through virtual check-ins and home visits. Given that their risk prediction scores did not meet the 50% threshold, additional intensive services were not provided. Changes in local policies promoting multi-month dispensing of ARVs to people living with HIV have resulted in the majority of those who are newly enrolled on ART being provided with a 90-day supply of medication and a smaller proportion provided with a 30-day supply of ARVs.

Of the 48 individuals who provided feedback on usability and acceptability, 36 (75%) indicated that the IIT prediction tool was useful. Common reasons they cited included early notification to the site of a client with high IIT potential and the ability to improve case management at the site, thus helping patient management and monitoring be more proactive than reactive. As one facility backstop mentioned:

It has helped us to monitor our clients, calling them up and giving them a timeline to come for their refills so that their treatment won't be interrupted.

While most data entry clerks and monitoring and evaluation specialists provided positive feedback on accessibility, a few were skeptical or neutral. Those with a positive view indicated that since the model was integrated into LAMIS, Nigeria’s routine national HIV information system, rather than a secondary application, it was straightforward and easy to navigate. One data entry clerk reported:

My experience using the machine learning predictive is that as a data entry clerk I will use the machine to check and relate with my case manager to track the client in time to avoid IIT.

A monitoring and evaluation specialist from a primary health center said:

At first, I found it challenging to understand the chance of IIT, but after understanding and using it, I now see it as indices to protect our program growth from negative adjustment.

One of the more skeptical data entry clerks related that:

I haven't seen to understand the logic behind it...The outcome didn't change the restart or return to care. I need the ideas behind this...

## Discussion

### Principal Results

Using routinely collected service delivery data, we developed an ML model to predict IIT among people living with HIV in Nigeria that was easy to introduce and acceptable to providers in routine clinical care settings. All models developed included the use of routinely collected individual- and clinic-level variables to determine the risk of IIT among clients receiving a 30-day supply of ART. The final model chosen had both sensitivity and positive predictive values higher than 80%. After initial challenges, our model was successfully incorporated into the national systems for routine individual-level case management and monitoring and evaluation in pilot clinics. We found health care workers to be amenable to incorporating the prediction tool into routine work and eager to increase opportunities to tailor interventions to those most in need. Our ML model performed well on our test data and integrated well into routine systems but has yet to be deployed and assessed for effectiveness at the population level.

### Limitations

The low number of clients receiving 30 days of ART limited our ability to make programmatic adjustments based on the likelihood of IIT and prevented the prospective assessment of performance or effectiveness. As multi-month scripting is now the norm, models incorporating the multi-month dosing data or developing a new model to be used among clients receiving 3 months or more of ART are needed. Additionally, more work is needed to understand the sensitivity and specificity of the model on IIT after the first 30 days and the usefulness of these models outside of the population or geography on which they were based.

The limitations that are inherent in routinely collected service delivery data will also need to be addressed before these data are used for developing ML models. In Nigeria, as in many countries, social and contextual community factors were not routinely collected in their national health management information system and thus were not factored into the model despite known associations with IIT. In our data set, we encountered high levels of missing and misclassified data that were handled statistically yet are illustrative of the challenges related to data quality. After the incorporation of the model into LAMIS, staff took greater care to address delayed and incomplete data entry, resulting in a significant reduction in the proportion of missing data. HIV programs have changed over time and continue to change quickly. Developing a model based on retrospective data is a limitation, and models must be tested prospectively to determine if the accuracy holds with newer data. As the wealth of programmatic data continues to grow, refining models as a tool to target services and improve the quality of care will be critical.

### Comparison With Prior Work and Implications

Using ML to improve continuity of HIV care is a practical example of how advanced analytics can address population- and individual-level global health challenges, as we continue to advance digital health maturity [14,21]. While ML analytics hold great promise for closing the final gaps to achieve the 95-95-95 targets, the representativeness of available and accessible data must be considered [15,22]. With representative data, ML models enable us to limit biases and increase service equity based on standard algorithms. In addition, ML models could be a useful tool that future programs could use to tailor interventions to a person’s unique needs. This can decrease differences in the quality of health care across sites or decrease the perpetuation of any health care worker bias against some vulnerable populations. From a sustainability perspective, addressing constraints in digital infrastructure and human resources are critical investments for scaling country-owned predictive analytics for addressing IIT and other important public health issues. Investments in this area can also contribute to the growth of a country’s broader digital health system architecture.

Recently, there have been increasing efforts in low- and middle-income settings to develop and integrate predictive analytics into public health programs and to demonstrate that these tools can be implemented in low-resource settings. In any setting where optimization is critical due to labor or fiscal shortages, ML can help target the efficient use of human and financial resources. However, it is critical to consider broader partnerships, deployment, and scaling of ML to ensure that ongoing investments are strategic and sustainable. Such solutions may require additional budgeting for foundational infrastructure (eg, connectivity, cybersecurity, cloud housing, data management, electricity access), along with the human resources and capacity building needed for ongoing independent program support. Determining when it is strategic to invest in ML given the broader investments required for sustainable ML and the wide range of HIV interventions available to improve treatment continuity will require assessing cost-effectiveness. Considering costing and evaluation methodologies and prioritizing investments that benefit the strengthening of broader digital infrastructure are opportunities to realize economies of scale and a greater return on investment.

### Conclusions and Next Steps

Despite initial challenges, we were able to successfully develop and deploy an ML model into LAMIS, Nigeria’s routine HIV information system. There was a high level of acceptance of the ML model among staff at the pilot facilities. Our model will be refined as additional data are made available; this includes expansion to include IIT in the context of multi-month dosing. The model will be assessed with prospective data to refine the appropriate cutoff for determining high risk and thus the threshold for providing additional services.

## Abbreviations

ART: antiretroviral therapy
ARV: antiretroviral
IIT: interruption in treatment
LAMIS: Lafiya Management Information System
ML: machine learning
PEPFAR: US President’s Emergency Plan for AIDS Relief
SIDHAS: Strengthening Integrated Delivery of HIV/AIDS Services
TB: tuberculosis
UNAIDS: United Nations Joint Programme on HIV/AIDS
